# RNA m^6^A modification regulates cell fate transition between pluripotent stem cells and 2‐cell‐like cells

**DOI:** 10.1111/cpr.13696

**Published:** 2024-07-01

**Authors:** Zhongqu Su, Yu Dong, Jiatong Sun, You Wu, Qingqing Wei, Yuwei Liang, Zhiyi Lin, Yujun Li, Lu Shen, Chenxiang Xi, Li Wu, Yiliang Xu, Yingdong Liu, Jiqing Yin, Hong Wang, Kerong Shi, Rongrong Le, Shaorong Gao, Xiaocui Xu

**Affiliations:** ^1^ College of Animal Science and Technology, Shandong Key Laboratory of Animal Bioengineering and Disease Prevention Shandong Agricultural University Tai'an Shandong China; ^2^ Clinical and Translational Research Center of Shanghai First Maternity & Infant Hospital, Frontier Science Center for Stem Cells, School of Life Sciences and Technology Tongji University Shanghai China

## Abstract

*N* ^6^‐methyladenosine (m^6^A) exerts essential roles in early embryos, especially in the maternal‐to‐zygotic transition stage. However, the landscape and roles of RNA m^6^A modification during the transition between pluripotent stem cells and 2‐cell‐like (2C‐like) cells remain elusive. Here, we utilised ultralow‐input RNA m^6^A immunoprecipitation to depict the dynamic picture of transcriptome‐wide m^6^A modifications during 2C‐like transitions. We found that RNA m^6^A modification was preferentially enriched in zygotic genome activation (ZGA) transcripts and MERVL with high expression levels in 2C‐like cells. During the exit of the 2C‐like state, m^6^A facilitated the silencing of ZGA genes and MERVL. Notably, inhibition of m^6^A methyltransferase METTL3 and m^6^A reader protein IGF2BP2 is capable of significantly delaying 2C‐like state exit and expanding 2C‐like cells population. Together, our study reveals the critical roles of RNA m^6^A modification in the transition between 2C‐like and pluripotent states, facilitating the study of totipotency and cell fate decision in the future.

## Graphical abstract

Su et al. show that RNA *N* ^6^‐methyladenosine (m^6^A) modifications are preferentially enriched in zygotic genome activation (ZGA) transcripts and MERVL with high abundance in 2C‐like cells and can facilitate the exit of the 2C‐like state. Inhibition of m^6^A effectors can increase the stability of ZGA transcripts and expand the population of 2C‐like cells.

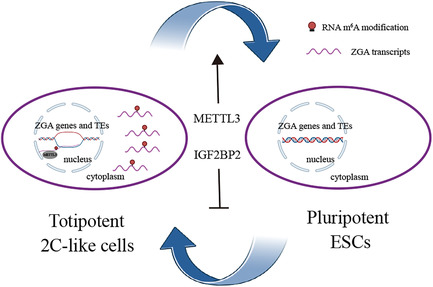

## 1 INTRODUCTION

Large‐scale and highly dynamic epigenetic and transcriptome remodelling occur after fertilisation and play important role in the establishment of totipotency.^1^ In mice, zygotic genome activation (ZGA) occurs in late 1‐cell and 2‐cell embryos (2C) which is closely linked with restoration of totipotency.^2^, ^3^, ^4^, ^5^, ^6^ ZGA needs to be tightly and precisely controlled as dysregulation of the ZGA transcripts results in embryo developmental failure and disease pathogenesis.^7^, ^8^, ^9^, ^10^

*N* ^6^‐methyladenosine (m^6^A) is the most abundant modification of messenger RNAs in eukaryotic cells, which is also found on repeat RNAs and long noncoding RNAs, and plays important roles in various developmental contexts.^11^, ^12^, ^13^, ^14^, ^15^, ^16^ Oocyte‐specific knockout of m^6^A reader YTHDF2 or IGF2BP2 leads to 2C embryo arrest in mice.^17^, ^18^ In addition, we have previously reported that blocking the function of m^6^A writer impairs the timely decay of ZGA transcripts.^19^ These studies highlight the important roles of RNA m^6^A modification in establishment of totipotency and cell fate decision in early embryos. However, the molecular details and the underlying mechanisms remain largely unknown due to the scarcity of totipotent cells in early embryos.

Mouse embryonic stem cells (ESCs) are capable of transiently entering a state with similar feature of 2C embryos.^20^, ^21^ In addition to showing similar 2C‐like transcriptome molecular features (such as reactivation of 2C marker genes and transposable elements [TEs]), 2C‐like cells also possess the capacity to contribute to both embryonic and extraembryonic tissues.^20^ Through the study of 2C‐like cells, a series of totipotency‐related regulators have been discovered.^22^, ^23^, ^24^, ^25^, ^26^, ^27^, ^28^, ^29^, ^30^, ^31^ Recently, different small molecular compounds have been used to induce totipotent‐like stem cells and construct blastocyst‐like structures with three germ layers in vitro.^32^, ^33^ However, the heterogeneity and highly dynamic nature in the transition between ESCs and 2C‐like cells greatly hinder the application of 2C‐like cells in research and medicine. Recent studies have found that depletion of m^6^A reader YTHDC1 is capable of greatly increasing the proportion of 2C‐like cells,^13^, ^34^ highlighting the role of RNA m^6^A modification in controlling cell fate transitions between 2C‐like cells and ESCs.

Using ultralow‐input RNA m^6^A immunoprecipitation (IP) developed by our lab recently,^19^ we mapped the transcriptome‐wide profiles of RNA m^6^A modification in 2C‐like cells and conducted detailed comparisons among 2C embryos, 2C‐like cells and ESCs. In general, RNA m^6^A modification was highly enriched in the ZGA transcripts with high abundance in 2C‐like cells. RNA m^6^A modification facilitates silencing of ZGA transcripts during 2C‐like–to–pluripotent state transition. In addition, inhibition of m^6^A methyltransferase METTL3 and m^6^A reader protein IGF2BP2 is capable of significantly delaying 2C‐like state exit and expanding 2C‐like cells population.

## 2 RESULTS

### 2.1 Transcriptome‐wide profiling of RNA m^6^A modification in 2C‐like cells

To investigate the transition between ESCs and 2C‐like cells, we utilised previously constructed and validated ESC lines containing MERVL‐tdtomato and p*Zscan4c*‐EGFP.^35^, ^36^ Next, we sorted 2C‐like cells (MERVL^+^/*Zscan4* ^+^) by fluorescence‐activated cell sorting and performed ultralow‐input RNA m^6^A IP as we developed previously to generate transcriptome‐wide m^6^A maps for 2C‐like cells.^19^ We used MACS2 to call m^6^A peaks (Table S1). 2C‐embryo‐specific transcripts, such as *Zscan4c*, *Usp17lb*, *Zfp352*, were upregulated in 2C‐like cells compared with ESCs, consistent with previous studies (Figure S1A).^20^, ^37^ Because we used *Glaussia luciferase* (GLuc) with m^6^A and *Cypridina luciferase* (CLuc) without m^6^A as spike‐in RNAs, we can calculate the ratio of GLuc/CLuc by quantitative polymerase chain reaction (qPCR) to monitor the enrichment efficiency of m^6^A antibody. The GLuc/CLuc ratio indicated that m^6^A transcripts in our library were highly enriched (Figure S1B). Then we generated the whole‐transcriptome m^6^A methylome profiles of 2C‐like cells, which included three replicates for IP and input libraries. Pearson correlation coefficiency analysis showed that replicates were highly comparable, and the global pattern of 2C‐like cells was more similar to ESCs than that of 2C embryos (Figure S1C). The distribution of m^6^A peak length was consistent with previous studies (Figure S1D).^38^, ^39^ In addition, de novo motif analysis of m^6^A peaks in 2C‐like cells identified the enrichment of canonical RRACH (R = G/A; H = A/U) motif (Figure S1E).^38^, ^39^

**FIGURE 1:**
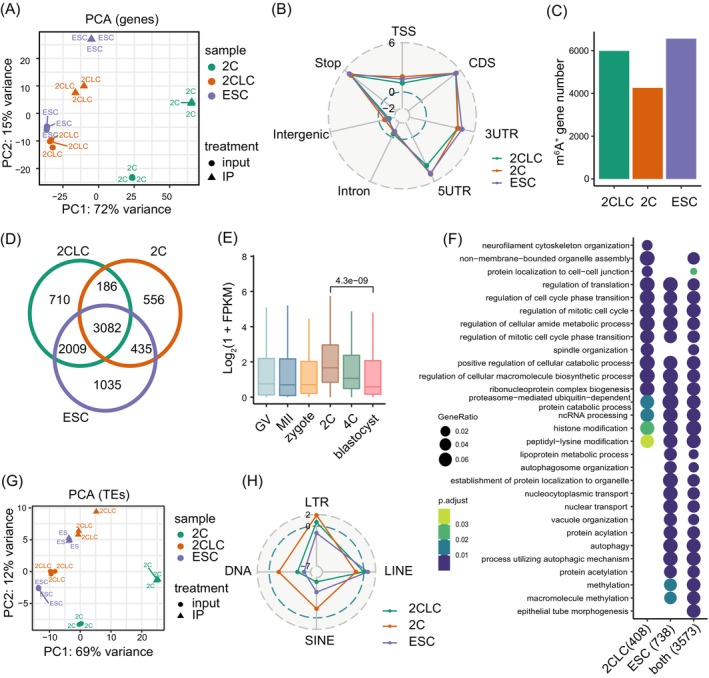
Transcriptome‐wide profiling of RNA m^6^A modification in 2C‐like cells. (A) Principal component analysis (PCA) of genes for m^6^A IP and input of 2C‐like cells (2CLC), 2C embryo (2C) and ESCs. (B) Radar chart showing the enrichment score (log ratio of observed/expected) of m^6^A peaks at TSS, 3′‐UTR, 5′‐UTR, CDS, intergenic regions, intron and stop codon. The enrichment score >0 indicates that m^6^A is enriched in the indicated regions. (C) Bar graphs showing the number of genes with m^6^A peak (m^6^A^+^) in 2CLCs, 2C and ESCs. (D) Venn diagrams displaying the overlap of m^6^A marked genes in 2CLCs, 2C and ESCs. (E) Box plot showing the expression levels of 186 genes with m^6^A in 2CLCs and 2C but without m^6^A in ESCs (Figure 1D) in pre‐implantation embryo development. Two‐tailed unpaired Wilcoxon test was used to calculate the *p* value. (F) GO analysis of m^6^A marked genes expressed in 2CLCs and ESCs specifically or both express. (G) PCA of TEs for m^6^A IP and input of 2CLC, 2C and ESC. (H) Radar chart showing the enrichment score (log ratio of observed/expected) of m^6^A peaks in four classes of TEs, including DNA transposons (DNA), SINEs, LINEs and LTRs. The enrichment score >0 indicates that m^6^A is enriched in the indicated regions.

We next performed detail analysis of the characteristics of RNA m^6^A methylation in 2C‐like cells. Principal component analysis (PCA) of coding genes based on the level of transcription and RNA m^6^A methylation showed that 2C‐like cells were similar to ESCs along the PC1 axis (constituting 72% of the variation), but along the PC2 axis (constituting 15% of the variation) 2C‐like cells were more similar to 2C embryos than ESCs (Figure 1A). Consistent with ESCs and 2C embryos, m^6^A methylation in 2C‐like cells was significantly enriched in coding sequences (CDSs), untranslated regions, and regions near stop codons (Figures 1B and S1F,G). The number of genes marked by m^6^A methylation in 2C‐like cells was slightly lower than that of ESCs, but higher than 2C embryos (Figure 1C). m^6^A‐marked transcripts preferentially displayed medium expression level in 2C‐like cells and ESCs while m^6^A‐marked transcripts in 2C embryos were enriched in genes with high transcriptional level (Figure S1H).

Next, we compared the m^6^A‐marked (m^6^A^+^) transcripts among 2C‐like cells, ESCs and 2C embryos. A large portion of m^6^A^+^ transcripts (5091 genes) was shared between 2C‐like cells and ESCs (Figure 1D). A total of 896 transcripts were specifically marked by m^6^A in 2C‐like cells compared with ESCs (termed as 2C‐like‐cell‐specific m^6^A transcripts) (Figure 1D). A total of 1470 m^6^A‐modified transcripts were specifically identified in ESCs compared with 2C‐like cells (termed as ESC‐specific m^6^A transcripts) (Figure 1D); 186 transcripts in 2C‐like cells acquired 2C‐embryo‐specific m^6^A modification (Figure 1D) and displayed highest transcriptional level at 2C stage during preimplantation development (Figure 1E; Table S2). Gene ontology (GO) analysis revealed that 2C‐like‐cell‐specific m^6^A transcripts were enriched in neurofilament cytoskeleton organisation, non‐membrane‐bounded organelle assembly, protein localization to cell–cell junction and spindle organisation (Figure 1F). ESC‐specific m^6^A genes were enriched in lipoprotein metabolic process, autophagosome organisation (Figure 1F).

Previous findings have shown that TEs transcripts were important in oocytes and early embryonic development,^40^, ^41^, ^42^ which were post‐transcriptionally controlled by m^6^A modifications.^16^, ^19^ PCA analysis of TEs based on RNA level and m^6^A intensity showed that 2C‐like cells were similar to ESCs along the PC1 axis (constituting 69% of the variation) (Figure 1G). m^6^A modification was prominently enriched in LTR RNAs in 2C‐like cells similar to 2C embryos (Figure 1H). In contrast, ESCs displayed enrichment of m^6^A on LINE RNAs rather than LTR RNAs (Figure 1H).

In summary, our results indicated that 2C‐like cells acquired partial RNA m^6^A modification signature of 2C embryos during 2C‐like transitions.

### 2.2 RNA m^6^A modification is correlated with the expression level of ZGA‐related transcripts in 2C‐like cells

**FIGURE 2:**
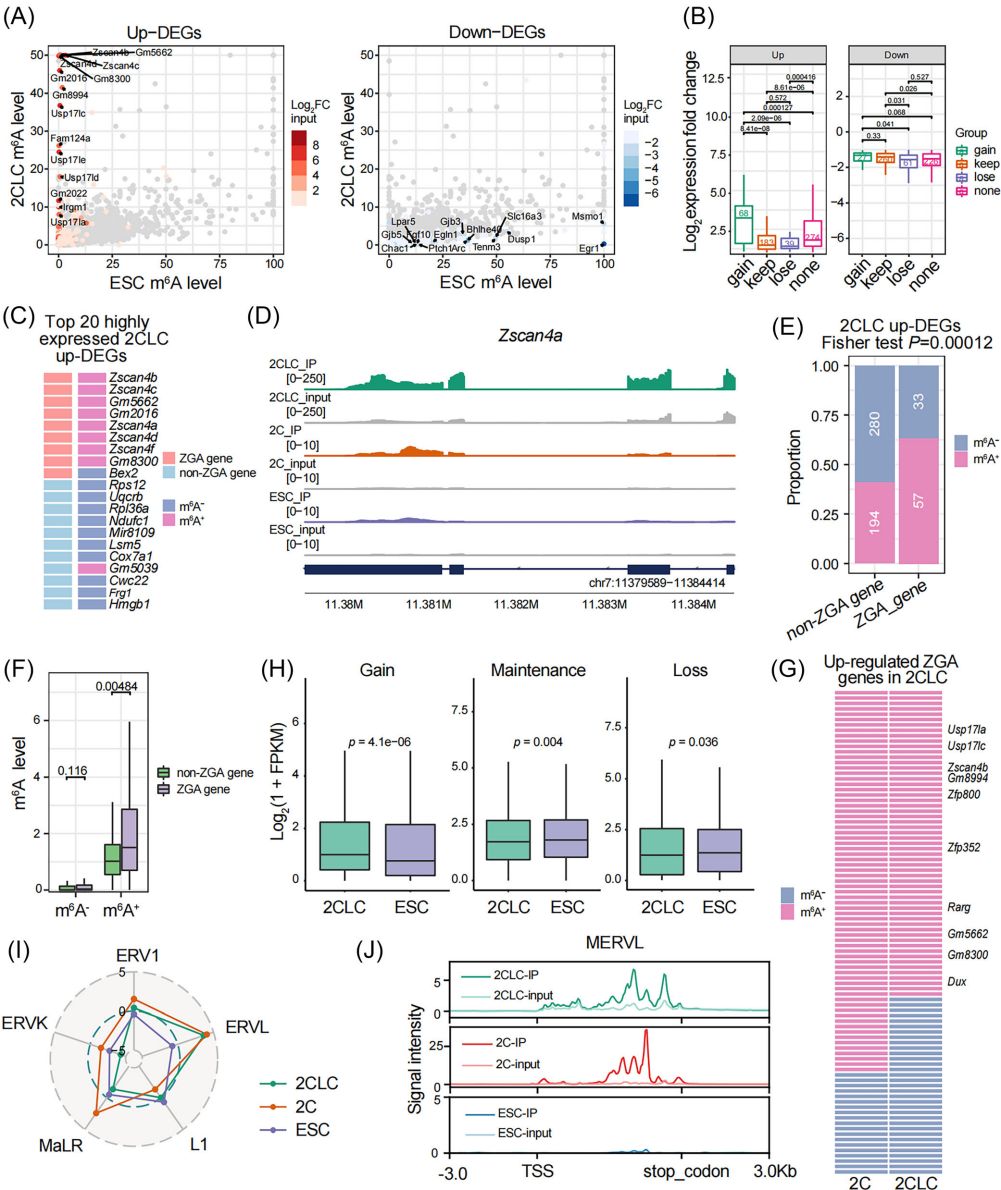
RNA m^6^A modification is highly enriched in ZGA transcripts and MERVL RNAs. (A) Scatter plots showing the relationship between m^6^A levels (calculated by m^6^A signal intensity) and differential gene expression. Red or blue plots indicate upregulated or downregulated differentially expressed genes (up‐DEGs or down‐DEGs, 2CLC/ESC), respectively. Grey indicates transcripts which are not differentially expressed. DEGs cut‐off is fold change (FC) >2 and FDR <0.05. (B) Box plot showing the expression fold changes (2CLC/ESC) of gain, keep, lose and none m^6^A transcripts in 2CLC compared with ESCs. Gain m^6^A represents 2CLC‐specific modified transcripts; keep m^6^A represents transcripts were both modified in 2CLCs and ESCs, lose m^6^A represents ESC‐specific modified transcripts and none m^6^A represents transcripts without modification in both 2CLCs and ESCs. (C) Heatmap showing whether the top 20 highly expressed up‐DEGs ranked by 2CLC FPKM (FC > 3) are ZGA genes and whether they are modified by m^6^A. (D) The UCSC browser track showing m^6^A IP and input reads of *Zscan4a*. (E) Bar graphs showing proportion and number of m^6^A^+^ and m^6^A^−^ transcripts in ZGA and non‐ZGA 2CLC up‐DEGs (2CLC/ESC). Two‐tailed Fisher's exact test was used to calculate the *p* value. (F) Box plot showing the m^6^A levels of m^6^A^−^ (left) and m^6^A^+^ (right) ZGA and non‐ZGA 2CLC up‐DEGs. (G) Heatmap showing whether the ZGA up‐DEGs in 2CLC or 2C are modified by m^6^A. (H) Box plot showing the expression levels of transcripts gained, maintained and lost m^6^A in 2CLCs and ESCs. (I) Radar chart showing the enrichment score (log ratio of observed/expected) of m^6^A peaks in major families of LINE and LTR. The enrichment score >0 indicates that m^6^A is enriched in the indicated TE family. (J) Average profile of m^6^A IP and input signal for full‐length MERVL copies in 2CLC. Two‐tailed unpaired Wilcoxon test was used to calculate the *p* values in B, F and H.

Next, we investigated the link between RNA m^6^A modification and transcriptional change during 2C‐like transition. Almost all the upregulated transcripts during 2C‐like transition were depleted for m^6^A in ESCs (Figure 2A, left). A portion of upregulated transcripts showed concomitantly elevated m^6^A levels in 2C‐like cells compared with ESCs (Figure 2A, left), indicating a potential link between RNA m^6^A modification and upregulated transcripts. Interestingly, the extensively upregulated 2C‐specific transcripts in 2C‐like cells, such as *Zscan4* family, *Gm8300* and *Gm2016* ^20^, ^37^ exhibited highest m^6^A levels (Figure 2A, left). Downregulated transcription in 2C‐like cells displayed a general trend of reduced m^6^A level, albeit to varying degrees (Figure 2A, right). We further classified differentially expressed genes (DEGs) between 2C‐like cells and ESCs into four categories according to the dynamics of m^6^A (Figure 2B). Among the four groups of upregulated transcripts, transcripts which gained m^6^A in 2C‐like cells displayed the highest level of activation (Figure 2B). In contrast, downregulation levels during 2C‐like transition were not correlated with m^6^A dynamics (Figure 2B).

Previous studies have showed that RNA m^6^A modification was preferentially enriched in highly expressed ZGA transcripts.^16^, ^19^ To investigate whether the positive correlation between transcriptional levels and RNA m^6^A modification was also existed during 2C‐like transition, we focused on the top 20 highly expressed upregulated transcripts in 2C‐like cells compared with ESCs, and found that almost all the ZGA transcripts belonging to this group were marked by m^6^A while m^6^A was absent in non‐ZGA transcripts (Figures 2C,D and S2A; Table S3). This finding indicated a close association between highly upregulated ZGA transcripts and RNA m^6^A modification in 2C‐like cells. Furthermore, the frequency of m^6^A^+^ transcripts was much higher in upregulated ZGA transcripts than the upregulated non‐ZGA transcripts in 2C‐like cells (Figure 2E), resembling the findings in 2C embryos as we previously reported.^19^ Besides, the upregulated m^6^A^+^ ZGA transcripts displayed higher levels of m^6^A intensity than m^6^A^+^ non‐ZGA transcripts (Figure 2F), indicating that m^6^A was preferentially deposited on ZGA transcripts during 2C‐like transitions (Figure S2B,C). The majority of upregulated ZGA transcripts in 2C‐like cells and 2C embryos displayed similar m^6^A deposition pattern with rare exception (Figure 2G). GO analysis revealed that upregulated transcripts modified by m^6^A were significantly enriched in process associated with response to virus while transcripts without m^6^A were significantly enriched in tumour necrosis process, such as regulation of tumour necrosis factor production (Figure S2D). We next explored the association between m^6^A dynamics and transcriptional change, during the transition from ESCs to 2C‐like cells (Figures 2H and S2E,F). Transcripts gained m^6^A peaks exhibited elevated expression levels while there were no significant expression changes in transcripts maintained or lost m^6^A peaks during 2C‐like transition (Figures 2H and S2E,F).

Murine endogenous retrovirus with leucine tRNA primer (MERVL) is the most highly transcribed retrotransposon in 2C embryos and 2C‐like cells (Figure S2G).^20^, ^42^ Interestingly, we also observed enrichment of m^6^A on MERVL in 2C‐like cells, resembling that of 2C embryos (Figures 2I,J and S2H).

In summary, RNA m^6^A modification is preferentially enriched in highly upregulated ZGA transcripts during 2C‐like transitions.

### 2.3 RNA m^6^A modification is associated with the rapidly reduced expression levels of ZGA transcripts during the exit of the 2C‐like state

**FIGURE 3:**
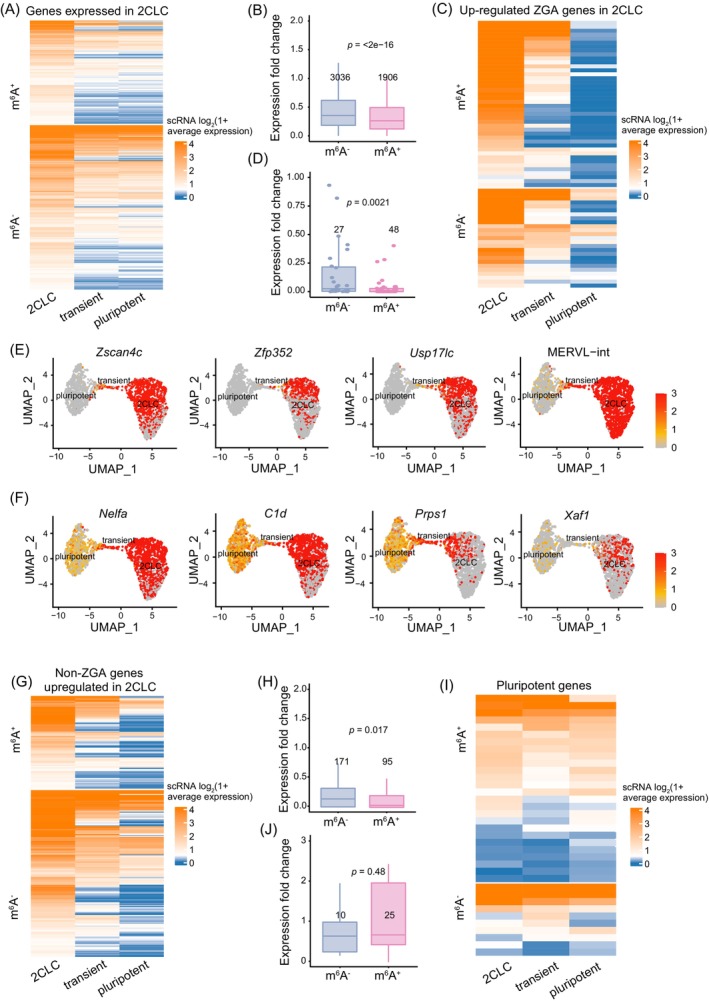
RNA m^6^A modification accelerates downregulation of ZGA transcripts and TEs during 2C‐like state exit. (A) Heatmap showing average expression level of single‐cell RNA‐seq clusters (GSE133234) in 2CLC‐to‐ESC transition. Genes are grouped by their m^6^A modification in 2C‐like cells. Cluster 2CLC (expression of 2CLCs marker genes), transient (expression of both 2CLCs and ESCs marker genes) and pluripotent (expression of ESCs marker genes) are distinguished by the expression of the marker genes. (B) Boxplot showing the expression fold change (cluster pluripotent vs. cluster 2CLC). The red and blue boxes represent the m^6^A^+^ and m^6^A^−^ gene expression fold change in panel A. The number of transcripts is marked at the top of the box. (C, G) Heatmap showing average expression level of single‐cell RNA‐seq clusters for ZGA (C) and non‐ZGA up‐DEGs (G) in 2CLC‐to‐ESC transition. Genes are grouped by their m^6^A modification in 2C‐like cells. (D, H) Boxplot showing the expression fold change (cluster pluripotent vs. cluster 2CLC) for ZGA (D) and non‐ZGA (H) up‐DEGs. The red and blue boxes represent the m^6^A^+^ and m^6^A^−^ gene expression fold change in panel C or G, respectively. The number of transcripts is marked at the top of the box. (E, F) UMAP showing the expression level of m^6^A^+^ (E) and m^6^A^−^ (F) ZGA up‐DEGs. All cells were colored by the relative expression level of the indicated genes. (I) Heatmap showing average expression level of single‐cell RNA‐seq clusters for pluripotent genes. Genes are grouped by their m^6^A modification in ESCs. (J) Boxplot showing the expression fold change (cluster pluripotent vs. cluster 2CLC) for pluripotent genes. The red and blue boxes represent the m^6^A^+^ and m^6^A^−^ gene expression fold change in panel I. The number of transcripts is marked at the top of the box. Two‐tailed unpaired Wilcoxon test was used to calculate the *p* values in B, D, H and J.

Previously, we have reported that RNA m^6^A modification facilitates the timely clearance of ZGA transcripts to ensure developmental progression of preimplantation embryos.^19^ Based on this finding, we speculated that RNA m^6^A modification may also regulate transcriptional dynamics during the transition from 2C‐like state to pluripotent state. To this end, we utilised a previously published single‐cell RNA‐seq data to further characterise m^6^A‐marked transcripts in this process.^43^ We first analysed the expression fold change of m^6^A^+^ and m^6^A^−^ transcripts of 2C‐like cells (Figure 3A,B). In general, m^6^A^+^ transcripts were more rapidly downregulated than m^6^A^−^ transcripts (Figure 3A,B). We then focused on upregulated ZGA transcripts in 2C‐like cells and found that m^6^A‐marked ZGA transcripts exhibited a much more accelerated downregulation rate than that of ZGA transcripts depleted with m^6^A in the process of 2C‐like‐to‐pluripotent state transition (Figure 3C,D; Table S4). Uniform manifold approximation and projection (UMAP) analysis further showed that m^6^A‐marked ZGA maker transcripts, including *Zscan4* family, *Usp17*‐like family, *Zfp352* and MERVL were substantially decreased preceding the activation of pluripotent genes (Figures 3E and S3A). In contrast, the downregulation of m^6^A^−^ ZGA transcripts was significantly slower than m^6^A^+^ ZGA transcripts, including *Nelfa*, *C1d* and *Xaf1* genes (Figure 3E,F). Together, these results suggest that m^6^A^+^ ZGA transcripts are preferentially to be rapidly decayed during exit of 2C‐like state.

To further investigate whether RNA m^6^A modification was also associated with other upregulated transcripts in 2C‐like cells, we next focused on non‐ZGA upregulated transcripts. We found that m^6^A^+^ non‐ZGA transcripts exhibited a slightly accelerated rate of downregulation than that of m^6^A^−^ counterparts (Figure 3G,H; Wilcox test *p* = 0.017), which is far less significant than the ZGA upregulated transcripts (Figure 3C,D; Wilcox test *p* = 0.0021). Next, we wondered whether pluripotent transcripts were associated with m^6^A methylation during 2C‐like‐to‐pluripotent state transition. However, we did not observe a significant difference in the expression change between m^6^A^+^ and m^6^A^−^ pluripotent transcripts (Figures 3I,J and S3B,C).

Given the important roles of RNA m^6^A modification, we next tried to identify factors involved in regulation of m^6^A methylation in 2C‐like cells. We compared the expression level of m^6^A‐related enzymes in 2C‐like cells and ESCs (Figure S3D–F; Table S5). *Igf2bp2*, which can stabilise target mRNA in an m^6^A‐dependent manner and plays an important role in ZGA, showed the most obvious transcriptional change during 2C‐like transition (Figure S3F).^18^, ^44^ Therefore, we explored the function of *Igf2bp2* in the subsequent analysis.

In summary, RNA m^6^A modification is associated with the rapid downregulation of ZGA transcripts during 2C‐like‐to‐pluripotent state transition.

### 2.4 RNA m^6^A modification promotes downregulation of ZGA transcripts and inhibition of IGF2BP2 can promote the transition of ESCs to 2C‐like cells

**FIGURE 4:**
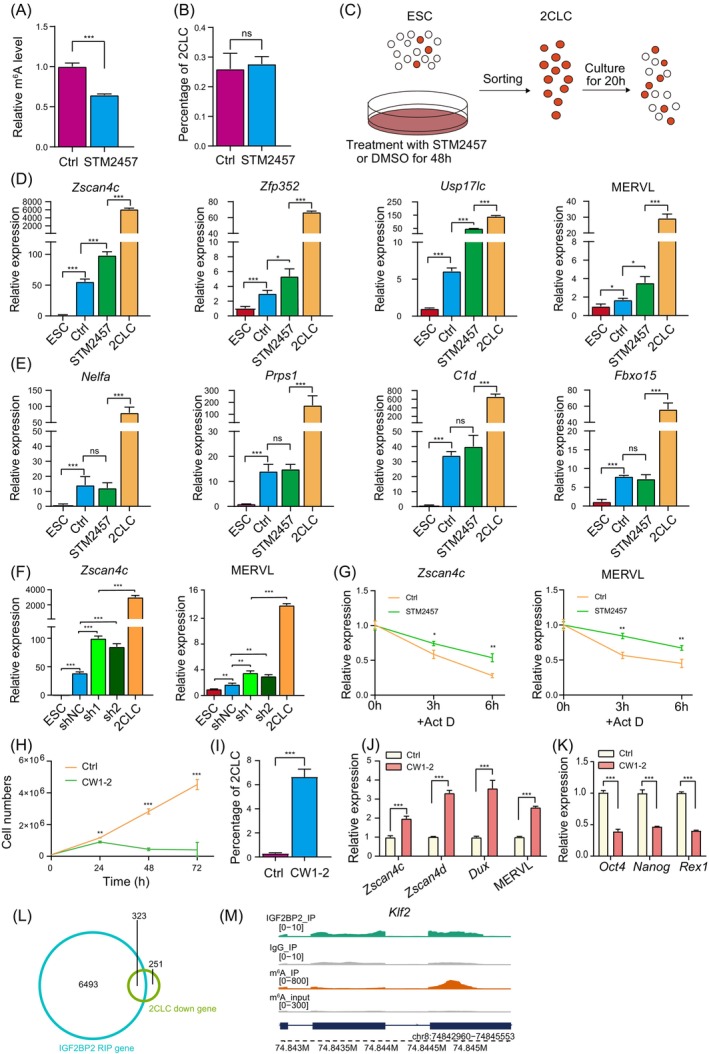
Inhibition of METTL3 and IGF2BP2, respectively, inhibits the degradation of ZGA transcripts with m^6^A and promotes the conversion of ESCs to 2C‐like cells. (A) Quantification of m^6^A abundance of the control (ctrl) and STM2457 treatment ESCs in Figure S4A. (B) Quantification of 2C‐like cell number in the control and STM2457 treatment ESCs in Figure S4C. (C) The workflow of the experimental process. The 2CLCs were isolated from 48 h DMSO or STM2457 treatment ESCs and were cultured in ESM with DMSO or STM2457 for 20 h. (D, E) RT‐qPCR detection of m^6^A^+^ (D) and m^6^A^−^ (E) ZGA genes in DMSO (marked as ctrl) or STM2457 (marked as STM2457) treated 2CLCs (Figure 4C). (F) RT‐qPCR detection of m^6^A^+^ ZGA genes in control shRNA (shNC, negative control) versus anti‐METTL3 shRNA (sh1, sh2) 2CLCs during 2C‐like state exit. (G) RT‐qPCR showing relative expression levels of *Zscan4c* and MERVL in the presence and absence of METTL3 inhibitor STM2457 at different times points. (H) Growth curves of ESCs after CW1–2 treatment. (I) Quantification of 2C‐like cells number in the control and CW1–2 treatment ESCs in Figure S4L. (J, K) RT‐qPCR detection of ZGA genes (J) and pluripotent genes (K) in control and CW1–2 treatment ESCs. (L) Venn diagrams displaying the overlap of IGF2BP2 target transcripts in ESCs and downregulated transcripts during the transition from ESCs to 2CLCs. (M) The UCSC browser snapshots showing signals of IGF2BP2 RIP‐seq, m^6^A RIP‐seq and input at *Klf2* loci in ESCs. Data in A, B, D–K was shown as mean ± SD; *n* = 3 biological replicates. Significance was analysed by using Student's *t*‐test (*p < 0.05; **p < 0.01; ***p < 0.001; ns, not significant).

To further investigate the role of RNA m^6^A modification during 2C‐like‐to‐pluripotent state transition, we used inhibitor STM2457 of METTL3 which is core factor of the m^6^A methyltransferase complex.^45^, ^46^ We first tested m^6^A level of ESCs after STM2457 treatment by dot blot (Figures 4A and S4A). We confirmed that m^6^A level was significantly reduced by 48 h following STM2457 treatment (Figures 4A and S4A). At this time, the frequency of 2C‐like cells (Figures 4B and S4B,C) and the expression level of 2C‐specific marker transcripts *Zscan4c* and MERVL were not significantly altered (Figure S4D). Given the aforementioned association between RNA m^6^A modification and downregulation of ZGA transcripts during 2C‐like state exit, we sorted 2C‐like cells in the presence or absence of STM2457 treatment, respectively (Figure 4C). We found that inhibition of m^6^A methyltransferase significantly delayed the downregulation of m^6^A‐marked ZGA transcripts, such as *Zscan4c*, *Zfp352*, *Usp17lc* and MERVL (Figure 4D). Furthermore, we did not observe any changes in the downregulation rate of ZGA transcripts depleted of m^6^A during 2C‐like state exit, such as *Nelfa* and *C1d* (Figure 4E), which was similar to our previous findings in 2C embryos.^19^ To further validate this result, we designed two independent short hairpin RNAs (shRNAs) to knockdown *Mettl3* in ESCs (Figure S4E). Similarly, knockdown of *Mettl3* led to impaired downregulation of ZGA transcripts during the exit of the 2C‐like state (Figure 4F), further supporting our findings for the roles of METTL3. To further confirm the role of RNA m^6^A in the degradation of ZGA transcripts, we first re‐analysed published m^6^A RIP‐seq data of METTL3 WT and KO ESCs and found that Mettl3 KO in ESCs significantly led to reduced m^6^A modification level on ZGA gene transcripts and MERVL (Figure S4F).^13^ This finding indicates that Mettl3 is a major regulator to establish m^6^A modification on ZGA transcripts. Based on these findings, we further performed RNA degradation experiment. Given the scarcity of spontaneously converted 2C‐like cells, we used our previously published strategy to induce 2C‐like cells by CRISPR‐mediated activation of MERVL (Figure S4G).^47^ We blocked transcription by actinomycin D treatment and analysed the influence of METTL3 and RNA m^6^A on the stability of ZGA transcripts. Inhibition of METTL3 via STM2457 significantly slows down the degradation rate of m^6^A‐modified ZGA transcripts (Figure 4G). These results indicate that METTL3 regulates the degradation of ZGA transcripts through m^6^A modification.

Given the significant expression change of IGF2BP2 in 2C‐like cells, we hypothesised that IGF2BP2 may function in 2C‐like transitions. In previous studies, IGF2BP2 has been shown to promote RNA stability in a m^6^A‐dependent manner in different contexts.^19^, ^44^ We induced expression of HA tagged IGF2BP2 under the control of doxycycline in ESCs (Figure S4H). Immunofluorescence staining indicated that IGF2BP2 was mainly distributed in the cytoplasm of ESCs (Figure S4I). Overexpression of IGF2BP2 resulted in slightly decreased cell growth rate of ESCs (Figure S4J), while there was no significant change in the expression levels of *Zscan4c* and MERVL (Figure S4K). Next, we used a reported small molecule CW1–2 to block the function of IGF2BP2.^48^ In the presence of CW1–2 treatment, proliferation rate of ESCs was significantly reduced (Figure 4H). Strikingly, the frequency of 2C‐like cells was considerably increased by 48 h CW1–2 treatment (Figures 4I and S4L). Consistently, the expression levels of ZGA marker transcripts, such as *Zscan4c*, *Zscan4d*, *Dux* and MERVL were significantly increased (Figure 4J), and the expression levels of pluripotent genes like *Oct4*, *Nanog* and *Rex1* were significantly decreased by 48 h CW1–2 treatment (Figure 4K). Thus, we speculated that IGF2BP2 may stabilise pluripotency‐related transcripts in ESCs and decreased IGF2BP2 may facilitate the transition from ESCs to 2C‐like cells. To validate this hypothesis, we performed RNA Immunoprecipitation Sequencing (RIP‐Seq) to investigate RNA bound by IGF2BP2. We found that the majority of downregulated transcripts during the transition from ESCs to 2C‐like cells are bound by IGF2BP2, including pluripotent genes *Klf2* (Figures 4L,M and S4M).

In summary, our results demonstrate that RNA m^6^A modification promotes the downregulation of ZGA transcripts during 2C‐like‐to‐pluripotent state transition and inhibition of IGF2BP2 is capable of significantly expanding 2C‐like cells population.

## 3 DISCUSSION

In the current study, we found that RNA m^6^A modification in 2C‐like cells is enriched in the ZGA transcripts whose expression levels are closely related to the intensity of m^6^A. During the exit of the 2C‐like state, RNA m^6^A modification promoted the downregulation of ZGA genes and MERVL. Of noted, inhibition of m^6^A methyltransferase METTL3 and m^6^A reader protein IGF2BP2 was capable of significantly delaying 2C‐like state exit and expanding 2C‐like cells population, indicating the important regulatory function of m^6^A in the cell fate transition between ESCs and 2C‐like cells.

In the transitions between ESCs and 2C‐like cells, RNA m^6^A modification is largely unchanged while only a small number of the transcripts undergoes m^6^A reprogramming with concomitant expression changes. RNA m^6^A modification is highly enriched in ZGA‐related transcripts with high abundance in 2C‐like cells, similar to that of 2C embryos.^19^ RNA m^6^A modification can promote maternal RNA degradation during the maternal‐to‐zygotic transition process in early embryos.^19^, ^49^ In addition, RNA m^6^A modification was also enriched in the ZGA genes with the high abundance in early embryos.^19^ The combined inhibition of METTL3, METTL14 and METTL16 prevented degradation of ZGA transcripts and thereby impaired embryonic development.^19^ By analysing published single‐cell RNA‐seq data and modulation of m^6^A methylation in 2C‐like cells, we demonstrated that RNA m^6^A modification can also promote the downregulation of ZGA transcripts during 2C‐like‐to‐pluripotent state transition.

Maternal deletion of IGF2BP2 results in decreased abundance of MTA RNA in oocytes and 2C embryos arrest in mice.^18^, ^19^ We found that the expression level of IGF2BP2 was decreased in 2C‐like cells compared with ESCs and inhibition of IGF2BP2 can expand 2C‐like cells population under normal ESCs culture conditions. A previous study has showed that IGF2BP2 was involved in the stabilisation of m^6^A‐marked transcripts in cancer cells.^44^ In ESCs, transcripts with high m^6^A intensity were enriched in the process of embryonic development.^50^ Based on these findings, we hypothesise that IGF2BP2 may control ESC‐to‐2C‐like cell transition by influencing the degradation of certain embryonic development transcripts marked by m^6^A modification. In addition, we also found decreased expression of YTHDC2 in 2C‐like cells compared with ESCs and its potential roles in the transitions between pluripotent and 2C‐like state need to be further explored.

## 4 CONCLUSIONS

In summary, our study provides a detail comparison of RNA m^6^A modification profiles among 2C embryos, 2C‐like cells, and ESCs. RNA m^6^A modification can facilitate the exit of the 2C‐like cell state. Inhibition of m^6^A effectors can increase the stability of ZGA transcripts and expand the population of 2C‐like cells. Therefore, our study may shed light on the mechanistic study of RNA m^6^A modification in the context of totipotency in the future.

## 5 MATERIALS AND METHODS

### 5.1 Ethics statement

The specific pathogen‐free mice were housed in the animal facility of Tongji University. All animal experiments were approved by the Animal Ethical and Welfare Committee of Tongji University under permit No.TJAB03224114.

### 5.2 Resource availability

Corresponding author: Further information and requests for resources and reagents should be directed to and will be fulfilled by the Lead Contact, Shaorong Gao.

Materials availability: All plasmids or mouse lines generated in this study are available from the lead contact with a completed Materials Transfer Agreement/without restriction.

### 5.3 Cell culture

Mouse embryonic fibroblasts (MEFs) were derived from 13.5‐dpc mouse embryo, and were cultured in DMEM (C11960500BT; Gibco) medium supplemented with 10% (vol/vol) fetal bovine serum (FBS) (10270‐106; Gibco) and 1 mM l‐glutamine (TMS‐002‐C; Merck Millipore). ESC lines with MERVL/Zscan4 double reporters had been reported^35^ were cultured on mitomycin C treated MEFs in ESM containing DMEM supplemented with 15% (vol/vol) FBS (16000‐44; Gibco), 1 mM l‐glutamine (TMS‐002‐C; Merck Millipore), 0.1 mM mercaptoethanol (ES‐007‐E; Merck Millipore), 1% nonessential amino acid stock (TMS‐001‐C; Merck Millipore) and 1000 U/mL LIF (ESGRO 1107; Merck Millipore). For STM2457 and CW1–2 treatment, we used 10 μM STM2457 or 1 μM CW1–2 in ESM medium and 1:2000‐diluted DMSO in ESM medium as a control. ESC cells were passaged and cultured on feeders in normal ESM medium, 24 h later replace with control or experimental medium. After another 48 or 72 h, the sample is collected or subjected to flow cytometry or cell count.

### 5.4 Reverse‐transcription PCR and quantitative PCR

For ESC samples, total RNA was extracted using RNAiso Plus reagent (9109; Takara), then was reverse transcribed using an All‐in‐One Reverse Transcription Kit (G490; ABM). For 2‐cell‐like cells (2C‐like cells) samples, cDNA were synthesised and amplified using Smart‐Seq2. Quantitative reverse‐transcription PCR (RT‐qPCR) was performed with SYBR Premix Ex Taq II and the ABI7500 Fast Real‐time PCR system (Applied Biosystems, Foster City, CA). The primer sequences are listed in Table S6. The reactions were performed in triplicate and relative mRNA expression was normalised to GAPDH as an endogenous control using the ΔΔ*C*_t_ method.

### 5.5 RNA decay

MERVL activated ESCs were cultured in ESM with 1 μg/mL puromycin (P9620; Sigma), 10 μg/mL Blasticidin S HCl (A11139‐03; Life Technologies), 250 μg/mL Zeocin (Z22100‐0.25; Thermo Fisher Scientific). Then cells were passaged to 12‐well plates and divided into two groups, one group added DMSO to the culture medium, and the other group added STM2457. Culture 48 h and added actinomycin D (10 μg/mL, A9415; Sigma), samples were collected at the corresponding time point for gene expression detection.

### 5.6 ULI‐MeRIP‐seq

For ULI‐MeRIP‐seq, 100 ng RNA were used per reaction, and three replicates were performed. Total RNA of 2‐cell‐like cells were extracted using PicoPure RNA Isolation Kit (KIT0204; Thermo Fisher Scientific). Upon assessment via Qubit RNA HS Assay (Q32852; Thermo Fisher Scientific), approximately 100 ng of total RNA was extracted from 10,000 2C‐like cells. The ULI‐MeRIP‐seq procedure was performed as previously described.^19^ To obtain m^6^A RNA, total RNA was interrupted by ultrasound and was incubated with 0.2–0.5 μL anti‐m^6^A antibody (NEB, E1610S)‐bead complex (protein A and protein G Dynabeads, Invitrogen) 4–5 h at 4°C. Library preparation of ULI‐MeRIP samples and total RNA samples was performed using a SMARTer Stranded Total RNA‐Seq Kit version 2 (634411; Takara) according to the manufacturer's protocol. The purified libraries were sequenced on an Illumina NovaSeq 6000 platform (Novogene and Nanjing Jiangbei New Area Biophamaceutical Public Service Platform).

### 5.7 Peak enrichment analysis

Enrichment of m^6^A peaks in gene‐related genomic elements and repeats elements was calculated as observed versus random counts. The observed counts were calculated as the occurrences of m^6^A peaks overlapping with related genomic regions using intersectBed of bedtools by restricting that the proportion of overlapped region is great than 50%. The random peaks were generated using shuffleBed. The random counts were calculated same as the observed counts. The radar plot for peak enrichment was plotted by R package ggradar.

### 5.8 GO analysis

GO analysis of genes was performed using compareCluster function of R package clusterProfiler (version 3.14.3)^51^ under parameters fun = “enrichGO,” pAdjustMethod = “BH,” ont = “BP,” OrgDb = org.Mm.eg.db, keyType = “SYMBOL,” qvalueCutoff = 0.05. Enrichment maps were constructed using R package Complexheatmap (version 2.2.0). GO terms for each functional cluster were summarised to a representative term.

### 5.9 Single‐cell RNA sequencing analysis

We downloaded filtered expression matrix from GSE133233, and processed the matrix using Seurat^52^ manually. Specifically, expression counts were scaled and log‐transformed using “NormalizeData()” function. The 500 most‐variable genes were identified by “FindVariableFeatures()” with parameters: selection.method = “vst,” nfeatures = 500. Then the data was scaled using “ScaleData().” PCA with 10 principal components were performed by “RunPCA(),” and UMAP dimension reduction with 8 principal components were performed by “RunUMAP.” A nearest‐neighbour graph using the eight dimensions of the PCA reduction was calculated using “FindNeighbors(),” followed by clustering using “FindClusters()” with a resolution of 0.2. Seurat's “DimPlot()” was used to plot cell clusters and “FeaturePlot()” function was used to demonstrate individual gene expression on UMAP embedding.

### 5.10 RIP‐seq

The RIP for IGF2BP2 protein was performed using ESCs according to manufacturer's instructions of Magna RIP™ RNA‐Binding Protein Immunoprecipitation Kit (17‐700; Merck Millipore). Each sample was IP with 4 μL rabbit anti‐IGF2BP2 (11601‐1; Proteintech) or IgG. The input and IP RNAs were extracted and subjected to library generation using KAPA Stranded RNA‐Seq Library Preparation Kit (KK8401; Roche).

### 5.11 Statistical analysis

R (version 3.6.1) was used for statistics analysis. Pearson's correlation coefficient was calculated using the cor.test function with default parameters to evaluate reproducibility of replicates. *p < 0.05; **p < 0.01; and ***p < 0.001. Fisher exact test was used for GO enrichment analysis by clusterProfiler. No statistical methods were used to predetermine sample size. Gene expression analysis was performed in GraphPad Prism version 8.0 (GraphPad Software, United States) using Student's *t*‐test. Data are presented as mean ± SD, and *p* values.

## AUTHOR CONTRIBUTIONS

R.L., X.X. and S.G. conceived and designed the experiments. Z.S. and Y.D. performed most of the experiments. X.X. performed the computational analysis. R.L., Z.S. Y.D. and X.X. designed and performed the data analysis. J.S., Y.W., Q.W., Y.L., Z.L., Y.L., L.S., C.X., Y.X., Y.L., J.Y., H.W. and K.S. assisted with the experiments. Z.S., R.L., X.X., and S.G. wrote the manuscript.

## CONFLICT OF INTEREST STATEMENT

The authors declare no conflict of interest.

## Supporting information


**Figure S1.** Validation of ULI‐MeRIP‐seq data quality in 2C‐like cells (2CLCs). Mainly related to Figure 1. (A) Scatterplots displaying the transcriptome comparison of 2CLCs and ESCs, upregulated and downregulated differentially expressed genes (up‐DEGs or down‐DEGs, 2CLC/ESC) are shown in red and blue, respectively. DEGs cut‐off is fold change (FC) >2 and FDR <0.05. (B) Bar plot showing high enrichment of m^6^A in 2CLCs IP samples tested by qPCR of GLuc versus CLuc. (C) Heatmap depicting the Pearson correlation of different samples of the top 2000 transcripts ranked by CVs of fold enrichment (IP/input) levels of m^6^A at ±200 bp around the stop codons. (D) Density of m^6^A peak length in 2CLCs, 2C and ESCs. (E) Sequence logo and *p* values of the consensus motif of m^6^A peak centres in 2CLCs. (F) Bar chart presenting the fraction of m^6^A peaks in different genomic regions. (G) Average profile of m^6^A IP and input signal of m^6^A^+^ genes in 2CLCs and ESCs. (H) Line chart displaying the relationship between m^6^A and gene expression in 2CLCs, 2C and ESCs.

**Figure S2.** m^6^A modification is enriched in ZGA transcripts and TEs. Mainly related to Figure 2. (A, B) The UCSC browser track showing m^6^A IP and input reads of *Zscan4d* (A) and *Dux* (B). (C) Average profile of m^6^A IP and input signal of m^6^A^+^ ZGA up‐DEG transcripts in 2CLCs. (D) GO analysis of up‐DEGs with or without m^6^A in 2CLCs. The number of genes is marked at the bottom of the box. (E, F) The UCSC browser track showing m^6^A IP and input reads of maintained (E) and lost (F) m^6^A transcripts examples. (G) Heat map of normalised RNA levels of transposon elements with RPM higher than 0.05 in at least one stage. (H) The UCSC browser track showing m^6^A IP and input reads of MERVL in 2CLCs and ESCs.

**Figure S3.** Dynamics of ZGA and pluripotent transcripts with or without m^6^A during 2C‐like state exit. Mainly related to Figure 3. (A) UMAP plot showing the expression level of ZGA transcripts example with m^6^A. (B, C) UMAP plot showing the expression level of pluripotent genes example with (B) or without m^6^A (C). (D–F) The expression of m^6^A writer (D), eraser (E) and reader protein (F) in 2CLCs and ESCs. *P* values was analysed by using Student's *t*‐test.

**Figure S4.** Inhibition of METTL3 and OE IGF2BP2 did not affect the number of 2CLCs. Mainly related to Figure 4. (A) m^6^A dot blot of the control (Ctrl) and STM2457 treatment ESCs. (B) Morphology of ESCs cultured in the presence and absence(Ctrl) of STM2457. Zscan4 expression was visualised with EGFP (green), and MERVL expression was visualised with tdTomato (red). Scale bar, 200 μm. (C) FACS analysis of mouse ESCs of MERVL:tdTomato cultured in control and STM2457 medium. (D) RT‐qPCR detection of ZGA genes in control and STM2457 treatment ESCs. (E) Expression levels of *Mettl3* in ESCs with control shRNA (shNC, negative control) versus anti‐METTL3 shRNA (sh1, sh2) by RT‐qPCR. (F) Average profile of m^6^A signal on ZGA gene transcripts and MERVL in WT and Mettl3 KO ESCs. (G)Relative expression of ZGA gene *Zscan4c* in MERVL‐activated ESCs compared with empty‐vector treated ESCs (Ctrl) by RT‐qPCR. (H) Western blot analysis showing overexpression (OE) HA tagged IGF2BP2. (I) Immunostaining analysis with HA antibody. Cell nuclei were visualised with DAPI. Scale bar, 20 μm. Two independent experiments were performed. (J) Growth curves of control and OE IGF2BP2 ESCs. Data are shown as mean ± SD (n = 3 independent wells). (K) RT‐qPCR detection of ZGA genes in control and OE IGF2BP2 ESCs. (L) FACS analysis showing frequency of 2C‐like cells cultured in the presence and absence of CW1‐2 treatment. (M) Distribution of IGF2BP2 RIP‐seq peaks in ESCs. Data in (D, E, G, J–K) was shown as mean ± SD; n = 3 biological replicates. Significance was analysed by using Student's *t*‐test (*p < 0.05; **p < 0.01; ***p < 0.001; ns, not significant).

**Table S1.** General statistics of the ULI‐MeRIP‐seq datasets generated in this study. Related to Figure S1.

**Table S2.** The expression levels of 186 m^6^A marked genes expressed in 2C‐like cells and 2C but not in ESCs in pre‐implantation embryo development. Related to Figure 1.

**Table S3.** Whether the upregulated ZGA genes of 2C‐like cells carries m^6^A compared with ESCs. Related to Figure 2.

**Table S4.** The downregulation rate of upregulated ZGA genes in 2C‐like cells compared with ESCs. Related to Figure 3.

**Table S5.** FPKM of m^6^A writer, eraser and reader protein in 2C‐like cells and ESCs RNA‐seq data. Related to Figure S3.

**Table S6.** Primer Sequences Used in this Paper. Related to Figure S1/4/S4.

**Data S1.** Supporting Information.

## Data Availability

The raw sequence data reported in this paper have been deposited in the Genome Sequence Archive (*Genom Proteom Bioinf* 2021) in National Genomics Data Center (*Nucleic Acids Res* 2022), China National Center for Bioinformation/Beijing Institute of Genomics, Chinese Academy of Sciences (GSA: CRA013820) that are publicly accessible at https://ngdc.cncb.ac.cn/gsa. Previously published MeRIP‐seq data of ESC that were re‐analysed here are available under accession code GSA: CRA003985. The public datasets we used for scRNA‐seq data can be accessed at NCBI GEO database under accession numbers GEO: GSE133234. The public datasets we used for MeRIP‐seq data of Mettl3 WT and KO ESC can be accessed at NCBI GEO database under accession numbers GEO: GSE146467.
