# Supplementary figures and images for: RNA m6A modification regulates cell fate transition between pluripotent stem cells and 2‐cell‐like cells
Source: Cell Prolif. 2024 Jul 1;57(9):e13696. doi: 10.1111/cpr.13696 (PMC11503247; doi:10.1111/cpr.13696)

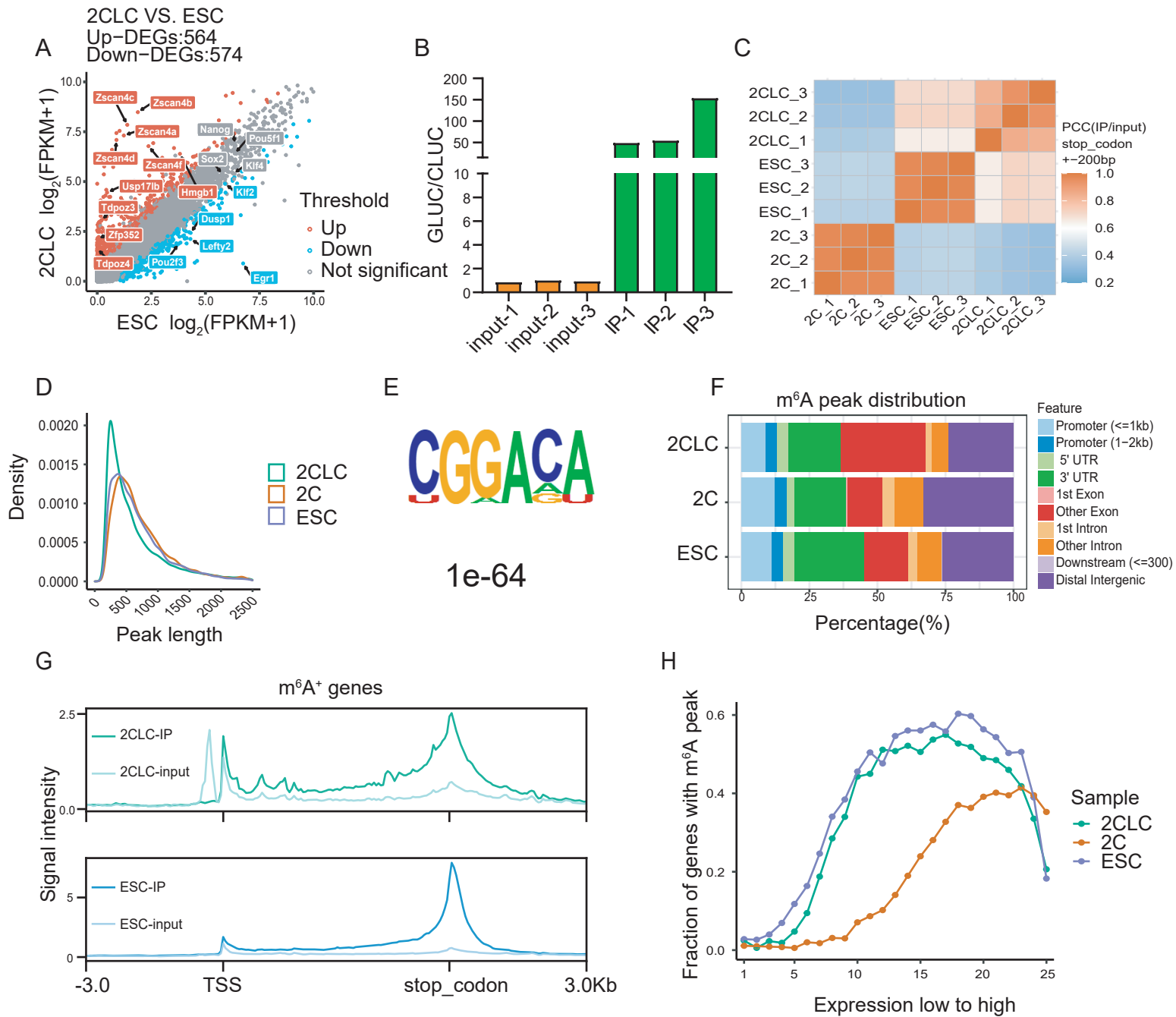

Figure S1

Supplement: Supplementary file 1 — Figure S1. Validation of ULI‐MeRIP‐seq data quality in 2C‐like cells (2CLCs). Mainly related to Figure 1. (A) Scatterplots displaying the transcriptome comparison of 2CLCs and ESCs, upregulated and downregulated differentially expressed genes (up‐DEGs or down‐DEGs, 2CLC/ESC) are shown in red and blue, respectively. DEGs cut‐off is fold change (FC) >2 and FDR <0.05. (B) Bar plot showing high enrichment of m6A in 2CLCs IP samples tested by qPCR of GLuc versus CLuc. (C) Heatmap depicting the Pearson correlation of different samples of the top 2000 transcripts ranked by CVs of fold enrichment (IP/input) levels of m6A at ±200 bp around the stop codons. (D) Density of m6A peak length in 2CLCs, 2C and ESCs. (E) Sequence logo and p values of the consensus motif of m6A peak centres in 2CLCs. (F) Bar chart presenting the fraction of m6A peaks in different genomic regions. (G) Average profile of m6A IP and input signal of m6A+ genes in 2CLCs and ESCs. (H) Line chart displaying the relationship between m6A and gene expression in 2CLCs, 2C and ESCs. [file CPR-57-e13696-s007.pdf]

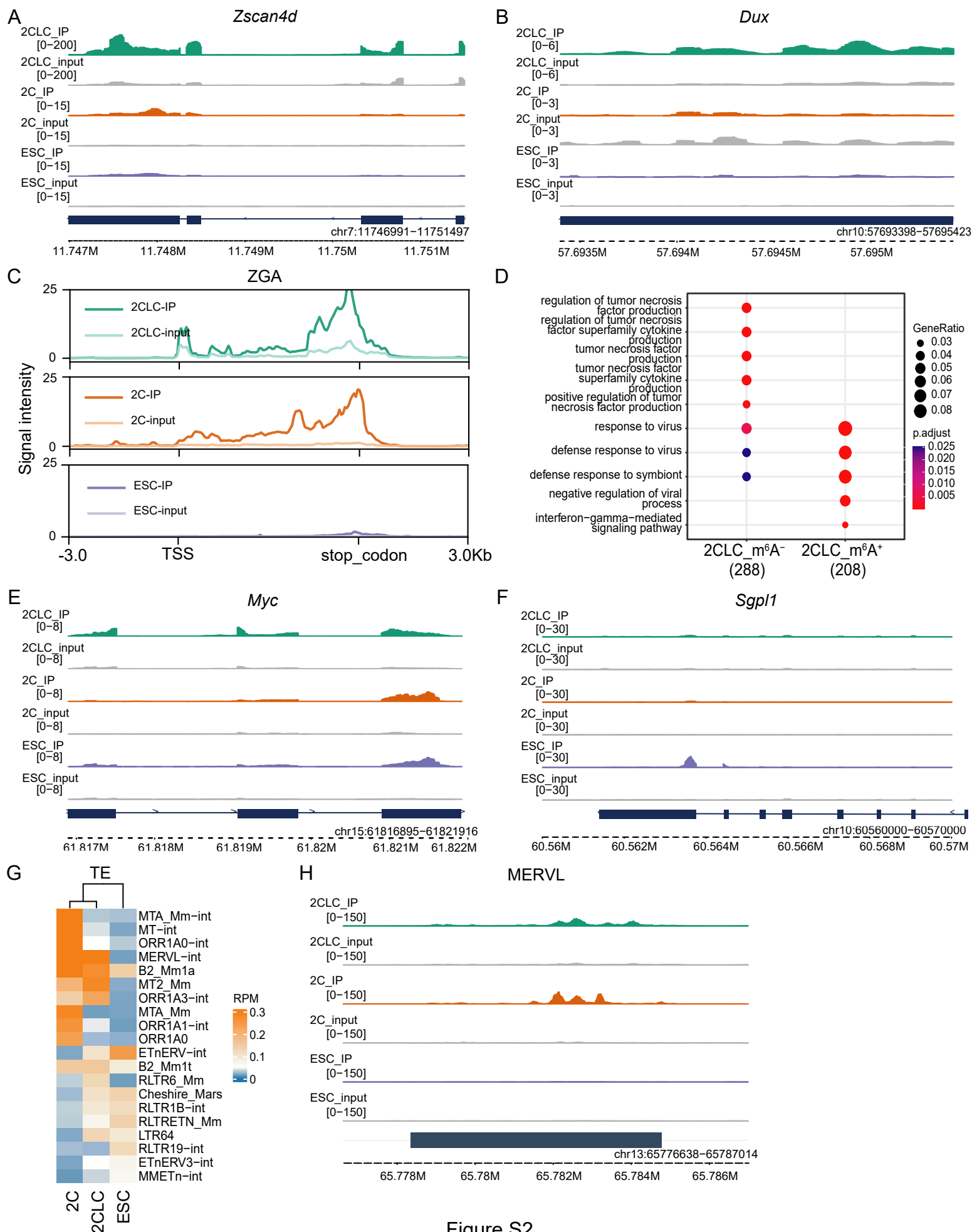

Figure S2

Supplement: Supplementary file 2 — Figure S2. m6A modification is enriched in ZGA transcripts and TEs. Mainly related to Figure 2. (A, B) The UCSC browser track showing m6A IP and input reads of Zscan4d (A) and Dux (B). (C) Average profile of m6A IP and input signal of m6A+ ZGA up‐DEG transcripts in 2CLCs. (D) GO analysis of up‐DEGs with or without m6A in 2CLCs. The number of genes is marked at the bottom of the box. (E, F) The UCSC browser track showing m6A IP and input reads of maintained (E) and lost (F) m6A transcripts examples. (G) Heat map of normalised RNA levels of transposon elements with RPM higher than 0.05 in at least one stage. (H) The UCSC browser track showing m6A IP and input reads of MERVL in 2CLCs and ESCs. [file CPR-57-e13696-s002.pdf]

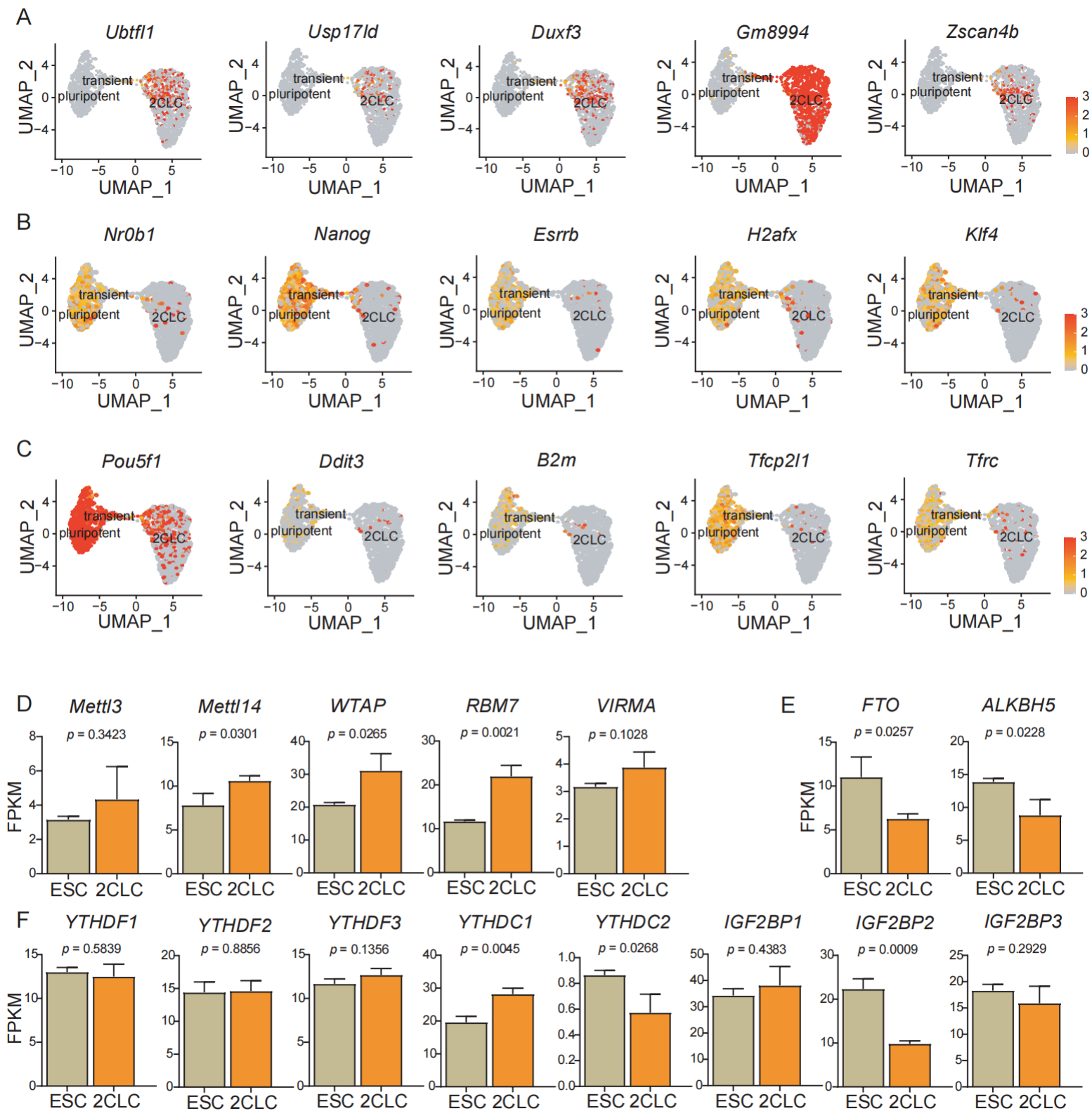

Figure S3

Supplement: Supplementary file 3 — Figure S3. Dynamics of ZGA and pluripotent transcripts with or without m6A during 2C‐like state exit. Mainly related to Figure 3. (A) UMAP plot showing the expression level of ZGA transcripts example with m6A. (B, C) UMAP plot showing the expression level of pluripotent genes example with (B) or without m6A (C). (D–F) The expression of m6A writer (D), eraser (E) and reader protein (F) in 2CLCs and ESCs. P values was analysed by using Student's t‐test. [file CPR-57-e13696-s005.pdf]

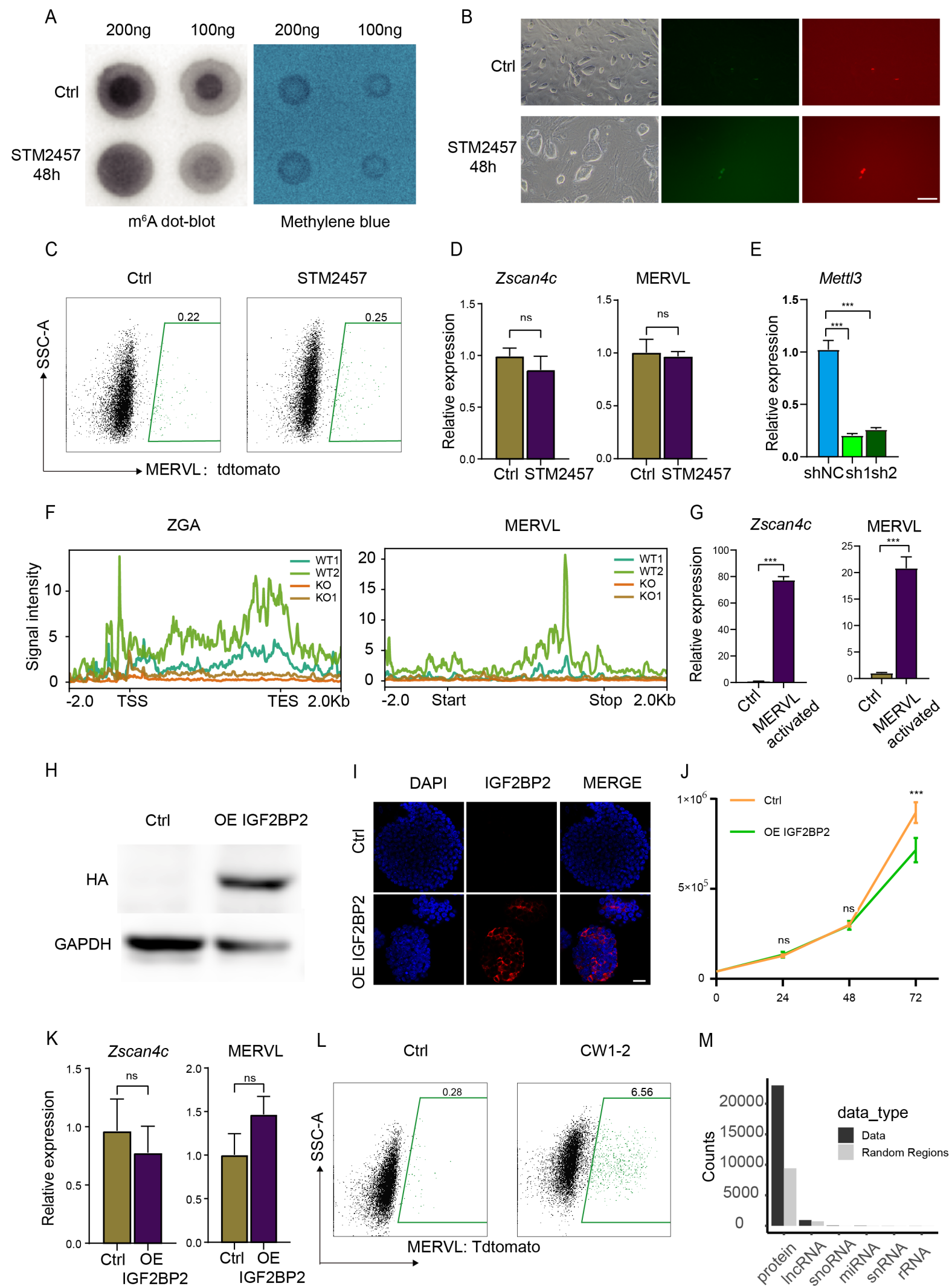

Figure S4

Supplement: Supplementary file 4 — Figure S4. Inhibition of METTL3 and OE IGF2BP2 did not affect the number of 2CLCs. Mainly related to Figure 4. (A) m6A dot blot of the control (Ctrl) and STM2457 treatment ESCs. (B) Morphology of ESCs cultured in the presence and absence(Ctrl) of STM2457. Zscan4 expression was visualised with EGFP (green), and MERVL expression was visualised with tdTomato (red). Scale bar, 200 μm. (C) FACS analysis of mouse ESCs of MERVL:tdTomato cultured in control and STM2457 medium. (D) RT‐qPCR detection of ZGA genes in control and STM2457 treatment ESCs. (E) Expression levels of Mettl3 in ESCs with control shRNA (shNC, negative control) versus anti‐METTL3 shRNA (sh1, sh2) by RT‐qPCR. (F) Average profile of m6A signal on ZGA gene transcripts and MERVL in WT and Mettl3 KO ESCs. (G)Relative expression of ZGA gene Zscan4c in MERVL‐activated ESCs compared with empty‐vector treated ESCs (Ctrl) by RT‐qPCR. (H) Western blot analysis showing overexpression (OE) HA tagged IGF2BP2. (I) Immunostaining analysis with HA antibody. Cell nuclei were visualised with DAPI. Scale bar, 20 μm. Two independent experiments were performed. (J) Growth curves of control and OE IGF2BP2 ESCs. Data are shown as mean ± SD (n = 3 independent wells). (K) RT‐qPCR detection of ZGA genes in control and OE IGF2BP2 ESCs. (L) FACS analysis showing frequency of 2C‐like cells cultured in the presence and absence of CW1‐2 treatment. (M) Distribution of IGF2BP2 RIP‐seq peaks in ESCs. Data in (D, E, G, J–K) was shown as mean ± SD; n = 3 biological replicates. Significance was analysed by using Student's t‐test (*p < 0.05; **p < 0.01; ***p < 0.001; ns, not significant). [file CPR-57-e13696-s001.pdf]
