# Supplementary material for: RNA m6A modification regulates cell fate transition between pluripotent stem cells and 2‐cell‐like cells
Source: Cell Prolif. 2024 Jul 1;57(9):e13696. doi: 10.1111/cpr.13696 (PMC11503247; doi:10.1111/cpr.13696)
Supplement: Supplementary file 11 — Data S1. Supporting Information. [file CPR-57-e13696-s009.docx]

**Supplemental Information**

**RNA m^6^A Modification Regulates Cell Fate Transition Between Pluripotent Stem Cells and 2-Cell-like Cells**

**Zhongqu Su^1,2^****^,3^, Yu Dong^2,3^, Jiatong Sun^2^, You Wu^2^, Qingqing Wei^1^, Yuwei Liang^2^, Zhiyi Lin^2^, Yujun Li^2^, Lu Shen^2^, Chenxiang Xi^2^, Wu Li, Yiliang Xu^1,2^, Yingdong Liu^2^, Jiqing Yin^2^, Hong Wang^2^, Kerong Shi^1^, Rongrong Le^2*^, Shaorong Gao^2,4*^ and Xiaocui Xu^2*^**

**SUPPLEMENTAL FIGURE LEGENDS**

**Figure S1. Validation of ULI-MeRIP-seq data quality in 2C-like cells (2CLCs). Mainly related to Figure 1.**

(A) Scatterplots displaying the transcriptome comparison of 2CLCs and ESCs, upregulated and downregulated differentially expressed genes (up-DEGs or down-DEGs, 2CLC/ESC) are shown in red and blue, respectively. DEGs cut-off is fold change (FC) >2 and FDR <0.05.

(B) Bar plot showing high enrichment of m^6^A in 2CLCs IP samples tested by qPCR of GLuc versus CLuc.

(C) Heatmap depicting the Pearson correlation of different samples of the top 2000 transcripts ranked by CVs of fold enrichment (IP/input) levels of m^6^A at ± 200 bp around the stop codons.

(D) Density of m^6^A peak length in 2CLCs, 2C and ESCs.

(E) Sequence logo and *p*-values of the consensus motif of m^6^A peak centers in 2CLCs.

(F) Bar chart presenting the fraction of m^6^A peaks in different genomic regions.

(G) Average profile of m^6^A IP and input signal of m^6^A^+^ genes in 2CLCs and ESCs.

(H) Line chart displaying the relationship between m^6^A and gene expression in 2CLCs, 2C and ESCs.

**Figure S2. m^6^A modification is enriched in ZGA transcripts and TEs. Mainly related to Figure 2.**

(A-B) The UCSC browser track showing m^6^A IP and input reads of *Zscan4d* (A) and *Dux* (B).

(C) Average profile of m^6^A IP and input signal of m^6^A^+^ ZGA up-DEG transcripts in 2CLCs.

(D) GO analysis of up-DEGs with or without m^6^A in 2CLCs. The number of genes is marked at the bottom of the box.

(E-F) The UCSC browser track showing m^6^A IP and input reads of maintained (E) and lost (F) m^6^A transcripts examples.

(G) Heat map of normalized RNA levels of transposon elements with RPM higher than 0.05 in at least one stage.

(H) The UCSC browser track showing m^6^A IP and input reads of MERVL in 2CLCs and ESCs.

**Figure S3. Dynamics of ZGA and pluripotent transcripts with or without m^6^A during 2C-like state exit. Mainly related to Figure 3.**

(A) UMAP plot showing the expression of ZGA transcripts example with m^6^A.

(B-C) UMAP plot showing the expression of pluripotent genes example with (B) or without m^6^A (C).

(D-F) The expression of m^6^A writer (D), eraser (E) and reader protein (F) in 2CLCs and ESCs. *P* values was analyzed by using Student’s t test.

**Figure S4. Inhibition of METTL3 and OE IGF2BP2 did not affect the number of 2CLCs. Mainly related to Figure4.**

(A) m^6^A dot blot of the control (Ctrl) and STM2457 treatment ESCs.

(B) Morphology of 2CLC cultured on feeder cells. Zscan4 expression was visualized with EGFP (green), and MERVL expression was visualized with tdTomato (red). Scale bar, 200 μm.

(C) FACS analysis of mouse ESCs of MERVL:tdTomato cultured in control and STM2457 medium.

(D) RT–qPCR detection of ZGA genes in control and STM2457 treatment ESCs.

(E) Expression levels of *Mettl3* in ESCs with control shRNA (shNC, negative control) versus anti-METTL3 shRNA (sh1, sh2) by RT-qPCR.

(F) Average profile of m^6^A signal on ZGA gene transcripts and MERVL in WT and Mettl3 KO ESCs.

(G)Relative expression of ZGA gene *Zscan4c* in MERVL-activated ESCs compared with empty-vector treated ESCs (Ctrl) by RT-qPCR.

(H) Western blot analysis showing overexpression (OE) HA tagged IGF2BP2.

(I) Immunostaining analysis with HA antibody. Cell nuclei were visualized with DAPI. Scale bar, 20 μm. Two independent experiments were performed.

(J) Growth curves of control and OE IGF2BP2 ESCs. Data are mean ± SD (n = 3 independent wells).

(K) RT–qPCR detection of ZGA genes in control and OE IGF2BP2 ESCs.

(L) FACS analysis of mouse ESCs of MERVL:tdTomato cultured in control and CW1-2 medium.

(M) Distribution of IGF2BP2 RIP-seq peaks in ESCs.

Data in (D-E, G, J-K) was shown mean ± SD; n = 3 biological replicates. Significance was analyzed by using Student’s t test. (* p < 0.05, ** p < 0.01, *** p < 0.001; ns, not significant.)

**SUPPLEMENTAL MATERIALS AND METHODS**

**Establishment of the METTL3 knockdown** and **inducible overexpression IGF2BP2 mESC line**

Small hairpin RNAs (shRNAs) for METTL3 knockdown were synthesized and subcloned into pSicoR vector . An HA tag fused to the N terminus of IGF2BP2 was constructed in the PCW57.1 vector. HEK293T cells were transfected with the KD or OE plasmid and the virus-packing plasmid psPAX2 and pMD2G using vigofect reagent. After 48 h, collect the medium with the virus and concentrate with 10% PEG8000 at 4 °C for 8-12 h. Centrifuge discarded the supernatant and resuspend with 200μl ESM, infecting 10,000 ESCs in a 96-well U-plate for 8-12 h. The infected ES cells were then cultured in ESM with feeder. Doxycycline hyclate (1 μg/mL) was used for the overexpression of exogenous genes in the PCW57.1 vector.

**Immunofluorescence Staining**

Cells were fixed with 4% paraformaldehyde overnight and were then permeabilized with 0.5% Triton X-100 in PBS for 15 min at room temperature. Next were blocked with 2.5% bovine serum albumin (BSA) in PBS at room temperature for 1 hour, and then incubated with HA primary antibodies (Cell Signaling Technology, 3724S,1:1000) overnight at 4°C. Washing three times with PBS, the samples were incubated with the secondary antibody at room temperature for 1 hour. Washing three times with PBS and staining the nuclei with DAPI. Using ZEISS LSM 880 microscope for imaging and signal intensity statistics were performed using ZEISS processing software.

**Western blot analysis**

∼2×10^6^pluripotent stem cells were collected and washed once with PBS and were lysed by adding 200 μL of lysis buffer on ice for half an hour and then ultrasonicated. Samples were then boiled to 100°C for10 minutes in loading buffer (EpiZyme, LT101S) with 2% β-mercaptoethanol (Amersham, CT). Collect the supernatant after centrifugation at 12,000 rpm at 4 °C for 5 min. Western blot experiments were performed following the Abcam protocol. Primary antibodies Anti-HA (1:1000; Cell Signaling Technology, 3724S) and Anti-GAPDH (1:1000; Proteintech, 60004-1-Ig) were incubated overnight at 4°C in continuous agitation. GAPDH was used as an endogenous loading control. HRP-labeled anti-mouse (1:2000; Beyotime, A0216) or HRP-labeled anti-rabbit antibodies (1:2000; Beyotime, A0208) were used as secondary antibodies. The signals were visualized by the ChemiDoc MP imaging system (Bio-Rad).

**m^6^A Dot blot assay**

Total RNA was isolated immediately after cells harvest using Trizol, and purified by Dynabeads® mRNA purification kit (Thermo Scientific, 61012). mRNA samples were quantified using UV spectrophotometry, and indicated amounts of mRNA were denatured at 95 °C for 5 min and loaded onto a Hybond-N^+^ membrane (Beyotime, FFN02) and cross-linked by UV. Then the membrane was blocked by 5% skimmed milk for 1h and incubated with m^6^A primary antibody (1:1000, NEB, E1610S) at 4°C overnight. After washing the membrane with TBS-T for 4 times, an ECL peroxidase-labeled anti-rabbit antibodies (1:2000; Beyotime, A0208) were used as secondary antibodies and incubated at RT for 1 h. The immunocomplex was photographed using the ECL imaging system. Finally, methylene blue staining quantifies the amount of mRNA. The signal density of the dot-blot experiment is quantified by image J software.

**RNA-seq data processing**

RNA-seq data were first subjected to Trim_galore (version 0.6.4) for adaptor trimming as well as quality control with the parameters --paired -j 7 --basename. The trimmed paired-end reads were then aligned to mm9 reference genome with random chromosome cleaned by STAR (Dobin et al., 2013) (version 2.7.11a) under the parameters --outSAMstrandField intronMotif --outFilterMultimapNmax 5000 --outSAMmultNmax 1 --outFilterMismatchNmax 999 --outFilterMismatchNoverLmax 0.04 . The expression of genes was quantified as FPKM by Cufflinks(version 2.2.1) (Trapnell et al., 2010). For the downstream data analyses, FPKM values were averaged for each gene between replicates. The RefSeq gene annotation files were downloaded from UCSC. For genes with multiple isoforms, the longest transcripts were selected. The R package DESeq2 (version 1.26.0) (Love et al., 2014) were used for gene differential expression analysis. Fold change > 2 and FDR < 0.05 were used as cutoff for down-regulated and up-regulated genes. Genome coverage bigwig files for UCSC genome browser were generated by deeptools (version 3.5.0) (Ramírez et al., 2016) bamCoverage with parameters –normalizeUsing CPM -bs 1. Genome coverage bigwig files for aggregation plot were generated by bamCoverage with parameters –normalizeUsing RPKM -bs 50. Aggregation plot for input signal was plotted by computeMatrix and plotProfile functions of Deeptools package.

**ULI-MeRIP data processing**

The strategy for sequencing reads trimming, mapping of ULI-MeRIP data was the same with that of RNA-seq. To identify m^6^A modified genes, R package MeTPeak (Cui et al., 2016) was used with following parameters PEAK_CUTOFF_FDR = 0.05,

WINDOW_WIDTH = 50, SLIDING_STEP = 10, MINIMAL_MAPQ = 0,

FOLD_ENRICHMENT = 2, REMOVE_LOCAL_TAG_ANOMALITIES=F. The m^6^A peaks were identified by MACS2 (version 2.2.4) (Zhang et al., 2008) callpeak tool with the corresponding input sample serving as control. MACS2 was run with options ‘-g mm --nomodel --keep-dup all’ for each replicate. For downstream analysis, a peak was kept if it was shared in all replicates. Genome coverage bigwig files for UCSC genome browser were generated by deeptools bamCoverage with parameters –normalizeUsing CPM -bs 1. Genome coverage bigwig files for aggregation plot were generated by bamCoverage with parameters –normalizeUsing RPKM -bs 50. Aggregation plot for input signal was plotted by computeMatrix and plotProfile functions of Deeptools package.

**Consensus motif identification within m^6^A peaks**

High confidence peaks with fold change greater than 4 were used to search the enriched motif for genes or repeats. The coordinates of peak-summit regions (50 nt upstream and downstream flanking the summit) of high confidence peaks were retrieved, and those peak-summit regions were mapped to the annotated genes or repeats in order to fetch the strand information by bedtools. Then the peak-summit regions with strand information were subjected to findMotifsGenome.pl of HOMER (Heinz et al., 2010) suite to identify motifs under parameters -rna -len 6.

**RIP-Seq Data Processing**

RIP-seq data were first subjected to Trim_galore for adaptor trimming as well as quality control with the parameters --paired -j 7 --basename. The trimmed paired-end reads were then aligned to mm9 reference genome with random chromosome cleaned by STAR under the parameters --outSAMstrandField intronMotif --outFilterMultimapNmax 5000 --outSAMmultNmax 1 --outFilterMismatchNmax 999 --outFilterMismatchNoverLmax 0.04 . The RIP peaks in each IP sample were identified by the MACS2 callpeak tool with the corresponding input sample serving as a control. MACS2 was run with the options -g mm–nomodel–keep-dup all for each replicate. For downstream analysis, a peak was kept if it was shared among all replicates. RIP bound genes were defined as genes in which any exon overlapped with a peak. Genome coverage bigWig files were generated by the deepTools bamCoverage tool with the parameters –normalizeUsing CPM -bs 1. Peak distribution was calculated through peak overlapping with different genome regions using bedtools. Peak profiling was performed using a bigwig files by R package trackplot.

**References**

Cui, X., Meng, J., Zhang, S., Chen, Y., and Huang, Y. (2016). A novel algorithm for calling mRNA m^6^A peaks by modeling biological variances in MeRIP-seq data. Bioinformatics *32*, i378-i385.

Dobin, A., Davis, C.A., Schlesinger, F., Drenkow, J., Zaleski, C., Jha, S., Batut, P., Chaisson, M., and Gingeras, T.R. (2013). STAR: ultrafast universal RNA-seq aligner. Bioinformatics *29*, 15-21.

Heinz, S., Benner, C., Spann, N., Bertolino, E., Lin, Y.C., Laslo, P., Cheng, J.X., Murre, C., Singh, H., and Glass, C.K. (2010). Simple combinations of lineage-determining transcription factors prime cis-regulatory elements required for macrophage and B cell identities. Mol Cell *38*, 576-589.

Love, M.I., Huber, W., and Anders, S. (2014). Moderated estimation of fold change and dispersion for RNA-seq data with DESeq2. Genome Biol *15*, 550.

Ramírez, F., Ryan, D.P., Grüning, B., Bhardwaj, V., Kilpert, F., Richter, A.S., Heyne, S., Dündar, F., and Manke, T. (2016). deepTools2: a next generation web server for deep-sequencing data analysis. Nucleic Acids Res *44*, W160-165.

Trapnell, C., Williams, B.A., Pertea, G., Mortazavi, A., Kwan, G., van Baren, M.J., Salzberg, S.L., Wold, B.J., and Pachter, L. (2010). Transcript assembly and quantification by RNA-Seq reveals unannotated transcripts and isoform switching during cell differentiation. Nat Biotechnol *28*, 511-515.

Zhang, Y., Liu, T., Meyer, C.A., Eeckhoute, J., Johnson, D.S., Bernstein, B.E., Nusbaum, C., Myers, R.M., Brown, M., Li, W., and Liu, X.S. (2008). Model-based analysis of ChIP-Seq (MACS). Genome Biol *9*, R137.
